# The role of well-child visits in detecting developmental delay in preschool children

**DOI:** 10.1186/s12887-023-04005-1

**Published:** 2023-04-18

**Authors:** M. Moser, C. Müllner, P. Ferro, K. Albermann, O. G. Jenni, M. von Rhein

**Affiliations:** 1 Child Development Center, University Children’s Hospital Zurich, University of Zurich (UZH), Zürich, Switzerland; 2 Children’s Research Center, University Children’s Hospital Zurich, University of Zurich (UZH), Zürich, Switzerland; 3 Center for Social Pediatrics, Cantonal Hospital Winterthur, Winterthur, Switzerland

**Keywords:** Well-child visits, Developmental delay, Pre-school children, Primary care physicians, Parents` hesitancy against early interventions

## Abstract

**Background:**

Early detection of developmental delay (DD) in preschool children is crucial for counselling parents, initiating diagnostic work-up, and starting early intervention (EI).

**Methods:**

We conducted a register study of all preschool children referred for EI in the Canton of Zurich, Switzerland, in 2017 (*N* = 1,785) and used an online survey among primary care physicians (PCPs, *N* = 271) to evaluate the care service of DD children.

**Results:**

PCPs accounted for 79.5% of all referrals by physicians and had correctly referred over 90% of the children in need of EI at an average age of 39.3 months (SD 8.9). In the survey, which represents 59.2% of all pediatricians and 11.3% of all general practitioners in the Canton, PCPs reported performing a mean of 13.5 (range 0–50, SD 10.7) well-child visits per week to preschool children and estimated well-child visits to be the most frequent type of consultation (66.7%) for the identification of DD. Parents’ hesitancy in accepting further evaluation or support were reported by 88.7%.

**Conclusions:**

Most preschool children with DD are identified in well-child visits. These visits represent an ideal opportunity for early detection of developmental impairment and initiation of EI. Carefully addressing parents’ reservations could reduce the rate of refusal, thus improving early support for children with DD.

## Introduction

Developmental delay (DD) is one of the most frequent disorders in early childhood, with a prevalence of about 15% [1, 2]. Children with DD may suffer from a variety of impairments that are likely to develop into multiple chronic and lifelong conditions, including intellectual disability, speech problems, socio-communicative deficits, sensory impairments, other physical complications, and behavioral and emotional disorders. A wide body of literature underlines the importance of early identification of affected children in order to counsel parents, enable diagnostic work-up, and initiate appropriate therapeutic support for infants with or at risk of DD [3, 4, 5, 6, 7, 8, 9, 10, 11]. Children diagnosed with DD have better health and educational outcomes if detected and treated at younger ages [12, 13]. Therefore, intervening early offers substantial long-term advantages and economic benefits for society [14, 15]. Children who participate in a developmental screening program are more likely to be identified with DD, referred to EI, and eligible for EI services in a timelier fashion than children who received surveillance alone [16]. However, despite improvements in rates of developmental screening, referral rates of children with developmental concerns to medical and developmental professionals and for initiation of developmental intervention remain low [17].

One important opportunity for detecting developmental delays, but also sensory impairments, and behavioral problems early are well-child visits, which involve systematic assessment of a child [18]. In fact, the guidelines from pediatric and family practitioners’ associations recommend regular well-child visits [17]. Swiss national recommendations endorse preventive well-child visits at one week, one, two, four, six, nine, and 12, 18, and 24 months, and at four years of age, performed by office-based pediatricians or general practitioners [19], which comprise a screening checklist, and tracking of growth and developmental milestones, which are documented in a booklet. Eight of these well-child visits before the age of 6 years are covered by mandatory health care insurance. However, no evaluation of well-child visits’ effectiveness in detecting DD in preschool children has been performed among primary care physicians (PCPs). Furthermore, it is unclear to what extent PCPs contribute to referring preschool children to EI, what proportion of such children subsequently receive therapy, and which obstacles against referral are encountered by PCPs.

In the Canton of Zurich, Switzerland, population 1.5 million, of whom 82,500 were aged 0–5 years in 2017, a centralized system of two Units for early Special Needs Education (USNE) determine individual needs for early special needs education and early speech therapy. These evaluations, and consequent enrollments for EI are either based on assessments by professionals using standardized tools (i.e., specific tests for language, cognitive, and behavior), or based on comprehensive information from previous professional assessments and observations. Thus, the USNE are responsible for evaluating the individual EI needs, approving enrollment for either early special needs education or early speech therapy, and assigning a defined number of granted hours for the respective EI. Once approved by the USNE, expenses for these respective EI are covered by the cantonal authorities without additional costs for the families. USNE Data is collected in a register of all preschool children referred for EI. Children can be referred to the USNE by PCPs, hospital-based specialists, therapists, and parents. Thus, the register allowed us to answer the following research questions: How many preschool children are referred to EI by PCPs and how many by other groups? How many children are correctly identified by PCP and receive EI? Which types of primary care consultations lead to the detection of developmental delay? Which obstacles do PCP encounter against referral for EI?

## Methods

We based our study on two data sources: first, we used data from the Canton-wide register of EI, which contains data of all preschool children referred for evaluation of EI needs in the year 2017. Anonymized data from the database was analyzed with respect to referrer, type of therapy (whether special needs education or speech therapy), age and sex of children, and type and extent of therapy assigned. Data were analyzed only if parents had not opted out of the scientific use of their data, a procedure that was recommended and permitted by the Cantonal Ethics Committee (BSEC-Nr. Req. 2016–00,774), and approved by the Cantonal Data Protection Officer. Of the 1,945 children referred for EI in 2017, 98 (5.0%) were excluded because parents objected to the use of their data for research and 62 (3.2%) due to missing data. Therefore, 1,785 children were included, with a total of 1,984 EI referrals. To investigate the role of PCPs and well-child visits in referring children with DD for EI in the Canton of Zurich, we compared the rates of children referred by PCPs to the rates of children entering the system through other referrals. Furthermore, we analyzed whether significant differences between children referred by PCPs and children enrolling for EI through other referrals were observable with respect to age and approval by the USNE. This approval served as a proxy for the correct identification of children in need of EI.

To evaluate the experiences of PCPs with well-child visits in the early detection of developmental delay, we conducted an online survey in January 2020 that was sent to all 1′479 PCPs in the Canton of Zurich. In total, 271 PCPs participated in the survey, representing 59.2% (129/218) of all office-based pediatricians and 11.3% (142/1,261) of all general practitioners. Participants were asked by email to complete an online questionnaire on well-child visits, recognizing and referring children with developmental delay, and the number of families with affected children who refused referral to EI. The survey contained closed and open-ended questions and was developed in collaboration with a focus group of experienced PCPs. In particular, we asked the following 4 questions: 1.) *“*On average, I perform ___ (number) well-child visits per week”; 2.) “I make the suspected diagnosis of a developmental delay [As a total of 100%]: A: % in well-child visits, B: % in sick-child visits, and C: % after medical referral by colleagues; 3.) “Number of children (< 5 years) per year with refusal of further diagnostics or special educational measures by the parents”; and 4.) “I note the following reasons for resistance from parents to further diagnosis/therapy [multiple response]: A: Fear of diagnosis (stigmatization), B: Parents do not recognize deficits, C: Parents recognize deficits but believe child is recuperating, D: Parents do not see the benefit of therapy, E: Parents have had bad experiences with siblings or other relatives, F: Parents have financial concerns, G: Cultural reasons + [comment], H: Other + [comment].” In this context, “cultural reasons” was chosen as an umbrella term describing causes of hesitancy in the context of the cultural background (e.g., different concepts of child rearing, fears of exclusion or stigmatization, language gaps).

All data were statistically analyzed in IBM SPSS Versions 25, and 26, and for the most part descriptive analyses were performed. The Mann–Whitney U and Chi-squared test was conducted for all group comparisons. A *P*-value below 0.05 was considered statistically significant.

## Results

We analyzed the data of 1,785 children referred to the USNE for EI in 2017. Some 74.7% of all referrals came from PCPs, specialists or hospital physicians (see Table 1), 25.3% from therapists, educators, or parents. Among the referrals by physicians, PCPs accounted for 79.5%. Children were referred to the USNE at an average age of 38.4 months (SD 10.8) for either speech therapy (n = 1,228, 68.8%), early special needs education (*n* = 358, 20.1%), or both (*n* = 199, 11.1%). The ages of children referred by therapists, educators, or parents did not differ significantly from those of children referred by PCPs, whereas children’s ages at referral were significantly lower in the group referred by hospital physicians. In average, the USNE granted 57.1 (SD: 22.1) hours of speech therapy, and 64.7 (SD: 27.0) hours of early special needs education. The rates of boys (67.8%) and girls (32.2%) referred did not differ significantly between groups (data not shown).

**Table 1:** Children with DD referred to early interventions grouped by referrer

**Referrer**	**Number of referrals** (*N* = 1,984)	**Age at referral** (months)	sig	**Approval rate**	sig
PCP﻿s^§^	59.4% (*n* = 1,179)	39.3 ± 8.9	Ref	93.8% (*n* = 69)	Ref
Hospital physicians^#^	15.3% (*n* = 304)	35.0 ± 13.7	***	92.1% (*n* = 23)	ns
Therapists, educators	22.6% (*n* = 449)	38.2 ± 12.7	ns	93.2% (*n* = 30)	ns
Parents	2.6% (*n* = 52)	39.7 ± 10.5	ns	97.9% (*n* = 1)	ns

Descriptive statistics on children referred by primary care physicians (PCPs), hospital-based pediatricians, therapists, or parents: number of referrals, mean age at referral, and approval rate. ^§^PCPs were either office-based pediatricians (*n* = 1,128) or general practitioners (*n* = 51), ^#^hospital physicians were developmental pediatricians (*n* = 148) or other hospital-based specialists (*n* = 156); Chi-squared tests comparing the ages and proportions of children referred by other groups to the values of the group referred by PCPs; Ref.: reference value for the group comparisons (*t* tests); Level of significance: *** < 0.001, ** < 0.01, * ≤ 0.05. The total of 1,984 entries results from double registrations for more than one therapy for 199 children

Approval rates by the USNE (as a proxy of correct identification of children with DD) were not significantly different between the different groups of referrers, and ranged between 89.6 and 97.9% (see Table 1).

**Fig. 1:**
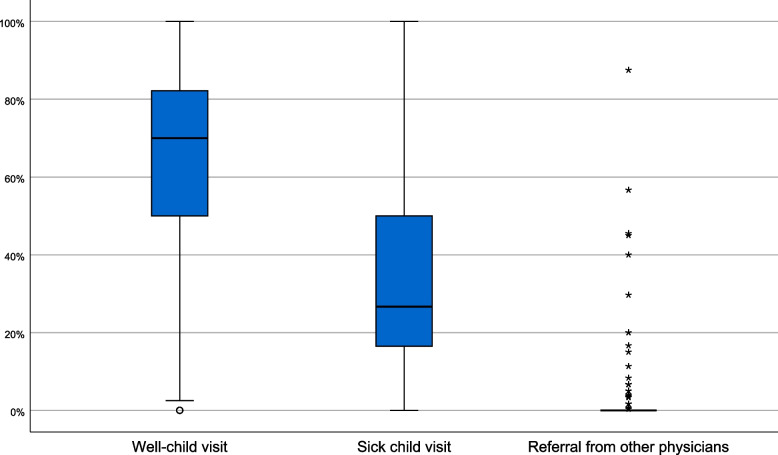
Box-plot of estimated proportions of consultation types leading to the diagnosis of developmental delay. Estimated percentages of initial reasons for consultations leading to a diagnosis of developmental delay: mental retardation, developmental language disorder, or autism spectrum disorder. Plots summarize responses from 148 participating PCPs who said they diagnosed at least one of these three types of developmental delay

The 271 PCPs participating in the survey were representative of all PCPs in the Canton of Zurich in age and gender (data not shown). They reported performing a mean of 13.5 (range 0–50, SD 10.7) well-child visits per week in preschool children. The PCPs were asked to estimate the percentage of well-child visits among all consultations in preschool children that lead to the diagnosis of the three most common developmental diagnoses: developmental language disorder, mental retardation, and autism spectrum disorder. Responding PCPs reported well-child visits to be the reason for the initial consultation (64.3%) twice as frequently as sick-child visits (32.5%), and referrals from other physicians (3.2%) were only mentioned occasionally (Fig. 1).

We also asked whether PCPs observed hesitancy among parents in accepting further evaluation and therapeutic support (“Number of children per year with refusal of further diagnostics or EI measures by the parents”). Some 88.7% of all PCPs reported observing this problem. The most common responses selected from the list of answers or described in free text were “parents believe that their child will recover the developmental delay”; “parents are afraid of the diagnosis”; “parents do not recognize the delay”; “parental stress”; “cultural reasons”; and “financial concerns”. Participants described serial appointments, a gentle (e.g., stepwise) confrontation with the child’s deficits, repeated explanations, and easy language, or the help of interpreters, or family members as strategies to convince parents.

## Discussion

The aim of this study was to investigate the role of PCP in the early identification of preschool children with DD. We found that most children are referred for EI by their PCPs: among the referrals by physicians, PCPs accounted for nearly 80%. This supports the importance of this group of health care professionals in detecting developmental delay and is in line with other work describing PCPs as the key players in identifying DD [20, 21] and in referring children for early intervention (Twardzik et al. [22]). This high importance of PCPs in detecting DD is also underlined by the high approval rate of referred children in this study, representing a high rate of children correctly screened as having DD. In our study, more than 90% of the children received EI after being referred to the USNE by PCPs, showing that in nearly all children, the in-depth assessment confirmed the clinical judgements of the PCPs. That no differences between the groups of referrers were found in approval rate or children’s age at referral might be explained by the close cooperation between these professional groups. For example, parents often consult their pediatricians before contacting the USNE. The high rate of approval in all groups therefore underlines the value of cooperation in networks at a high professional level. The only significant difference between referrers was that hospital-based specialists referred children significantly earlier to the USNE. This is most likely because referrals from hospital-based specialists generally include patients with medical conditions such as premature birth, severe illnesses, and conditions associated with developmental delay, which become apparent early in life.

Finally, we examined the views of PCPs on the role of well-child visits in detecting DD and their experiences with referring affected preschool children to EI. Physicians reported performing an average of 13.5 well-child visits per week to preschool children, indicating a high level of routine. They estimated detecting DD in preschool children twice as often in well-child visits as in sick-child consultations. We conclude that well-child visits are a valuable tool for early identification of developmental delay. This is consistent with King et al.’s findings that one important factor in helping to ensure early identification of DD is to offer easy access to well-child visits [23]. As in many countries, office-based PCPs in Switzerland are responsible for outpatient care of pre-school children, and well-child visits comprise over one quarter of their consultations [24, 25].

Surprisingly, nearly 90% of PCPs in our study described observing hesitancy among parents in accepting further evaluation and therapeutic support. This phenomenon has not been discussed extensively in the literature. Marshall et al. described how the referral process may be disrupted and how therapy advice does not always lead to EI [26]. In their study, the reasons for refusal were that parents overestimated their child’s performance or believed that the child would recover the developmental delay. This was also mentioned as one explanation by PCPs in our survey. Other reasons were linked to a lack of understanding what EI is, lack of knowledge about the system of care, or parental stress. Thus, it seems important to think of well-child visits as more than merely an opportunity for early detection of developmental delay. This type of consultation also offers the opportunity to inform parents about the care system, alleviate their concerns, and build a relationship with the family, so that difficult issues such as a DD and appropriate support options may be directly addressed. Carefully addressing parents’ reservations may reduce the rate of refusal and thus improve support for children with DD and their families. Accordingly, the PCPs in our study reported that one of their strategies in such cases is to arrange several appointments in order to convince parents gradually of the benefits of EI. This might also partly explain the average age of referral of 39.3 months in our cohort, which is quite late given the aim of an early identification of children with DD, and timely initiation of EI. It would certainly be interesting to investigate if mandatory pediatric visits before or at school entry as in Germany and Austria are helpful with regards to these concerns.

This study has strengths and limitations. Our results derive from a complete register of all EI measures (i.e., early special needs education, and early speech therapy) in the Canton of Zurich; this represents approximately 18% of the Swiss population in a region with health infrastructure comparable to many western industrial countries. We were able to achieve a high participation rate of PCPs in the survey. Unfortunately, we could not obtain objective numbers of well-child visits per participating PCP and had to rely on self-reports. Furthermore, we did not use psychometrically validated instruments for the survey but based the questionnaire on the expert opinions of a focus group of practitioners. Furthermore, the rate of participating general practitioners was much lower than the one of pediatricians. This might be due to the fact that we explicitly asked for the frequency of performed well-child visits in preschool children, which are much less commonly performed by general practitioners. However, we did not find significant differences between professional groups with respect to the key findings of our study. We therefore decided to treat all participants as one group of PCPs. It would have been interesting to know more about the participating PCPs` patients` characteristics, and if they are representative for the general population. Unfortunately, we only obtained strictly anonymized data from the participating primary care physicians (e.g., no postal codes), and no data on the SES, educational achievements, or native languages of their patients. The same data protection issues also forbid assessing the sensitivity of well-child visits to detect DD.

Finally, we can only speculate on the number of children with DD, who are missed by the system in place, since we don`t have data on the exact number of preschool children visiting PCP, and the number of children identified after kindergarten entry. However, based on prevalence numbers in the literature, we assume that the real number of preschool children with DD are higher. Thus, we are currently conducting a consecutive study which compares the rates of detection of DD in preschool children with the ones at school age.

We conclude that well-child visits represent an ideal opportunity for early detection of developmental impairments and for ensuring that EI, when required, can begin as early as possible. PCPs are key players in detecting children with DD and referring them for EI. PCPs can also use well-child visits to consult parents on child development issues and possible interventions.

## Data Availability

The datasets generated and/or analysed during the current study are only in part publicly available due to data protection regulations in Switzerland. To the extent, which is legally feasible, they are available from the corresponding author on reasonable request.

## Abbreviations

DD: Developmental delay
EI: Early intervention
PCP: Primary care physicians
SD: Standard deviation
USNE: Units for early Special Needs Education
